# microRNA-135b expression silences Ppm1e to provoke AMPK activation and inhibit osteoblastoma cell proliferation

**DOI:** 10.18632/oncotarget.15477

**Published:** 2017-02-18

**Authors:** Zheng-Wei Li, Yun-Rong Zhu, Xiao-Zhong Zhou, Bao-Biao Zhuo, Xiao-Dong Wang

**Affiliations:** ^1^ The Center of Diagnosis and Treatment for Children's Bone Diseases, The Children's Hospital Affiliated to Soochow University, Suzhou, China; ^2^ Department of Orthopedics, The Affiliated Jiangyin Hospital of Medical College of Southeast University, Jiangyin City, China; ^3^ Department of Orthopedics, The Second Affiliated Hospital of Soochow University, Suzhou, China; ^4^ Department of Orthopedics, The First People's Hospital of SuQian, SuQian, China

**Keywords:** microRNA-135b, Ppm1e, AMPK, osteoblastoma, cell proliferation

## Abstract

Forced-activation of AMP-activated protein kinase (AMPK) can possibly inhibit osteoblastoma cells. Here, we aim to provoke AMPK activation via microRNA silencing its phosphatase Ppm1e (protein phosphatase Mg^2+^/Mn^2+^-dependent 1e). We showed that microRNA-135b-5p (“miR-135b-5p”), the anti-Ppm1e microRNA, was significantly downregulated in human osteoblastoma tissues. It was correlated with Ppm1e upregulation and AMPKα1 de-phosphorylation. Forced-expression of miR-135b-5p in human osteoblastoma cells (MG-63 and U2OS lines) silenced Ppm1e, and induced a profound AMPKα1 phosphorylation (at Thr-172). Osteoblastoma cell proliferation was inhibited after miR-135b-5p expression. Intriguingly, Ppm1e shRNA knockdown similarly induced AMPKα1 phosphorylation, causing osteoblastoma cell proliferation. Reversely, AMPKα1 shRNA knockdown or dominant negative mutation almost abolished miR-135b-5p's actions in osteoblastoma cells. Further *in vivo* studies demonstrated that U2OS tumor growth in mice was dramatically inhibited after expressing miR-135b-5p or Ppm1e shRNA. Together, our results suggest that miR-135b-induced Ppm1e silence induces AMPK activation to inhibit osteoblastoma cell proliferation.

## INTRODUCTION

Osteoblastoma is one of the major causes of cancer-related mortalities among children and young teenagers around the World [1–4]. Remarkable improvements have been achieved in the diagnosis and clinical treatment for osteoblastoma, and the five-year overall survival has been increased to over 70% [1–4]. However, for those with advanced, malignant and/or recurrent osteoblastoma, the prognosis is still very poor [1–4]. Therefore, our group has been dedicated to understanding the pathologic mechanisms of osteoblastoma tumorigenesis and progression, and developing novel anti-osteoblastoma agents [5, 6].

Recent studies have proposed a pivotal function of AMP-activate protein kinase (AMPK), the key energy sensor kinase [7], in regulating cell survival and death. It has been shown that AMPK activation could also inhibit cancer cells via regulating its downstream targeting proteins [8–12]. AMPKα1 subunit Thr-172 phosphorylation is vital for AMPK activation [7, 13, 14]. The underlying mechanism of phosphorylation of this site has been extensively studied [15]. Several AMPK upstream kinases, including LKB1 [14], CaMKK [16] and TAK1 [17], have been identified thus far. Yet, the phosphatase de-phosphorylates AMPKα1 is largely unknown. Recent studies have proposed that protein phosphatase Mg^2+^/Mn^2+^-dependent (Ppm) 1e (Ppm1e) could possibly a key AMPKα1 phosphatase [18–20]. Ppm1e silence, inhibition and mutation were shown to induce AMPK activation via provoking AMPKα1 phosphorylation at Thr-172 [18–20].

microRNAs (miRNAs) are a group of endogenous small non-coding regulatory RNAs, which could inhibit gene expression at both translational and post-transcriptional levels [21, 22]. miRNAs bind to the 3′ untranslated region (UTR) of the targeted genes, causing mRNA decay as well as translational inhibition [21, 22]. Recent studies have identified a Ppm1e-targeting miRNA: miR-135b-5p [19, 20]. In the current study, we showed that miR-135b-5p expression silences Ppm1e, which activates AMPK to inhibit osteoblastoma cell proliferation.

## RESULTS

### miR-135b-5p upregulation correlates with Ppm1e upregulation and AMPKα1 de-phosphorylation in human osteoblastoma tissues

**Figure 1:**
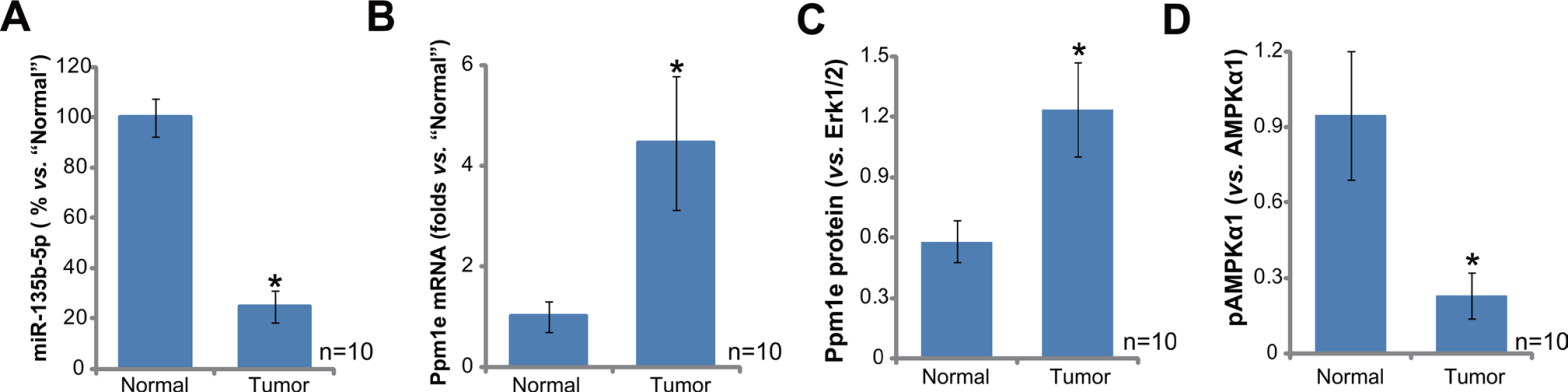
miR-135b-5p upregulation correlates with Ppm1e upregulation and AMPKα1 de-phosphorylation in human osteoblastoma tissues Expressions of miR-135b-5p (**A**, qRT-PCR assay), Ppm1e mRNA (**B**, qRT-PCR assay) and listed proteins (C–D, Western blotting assay) in ten (10) different human osteoblastoma tissues (“Tumor”) and surrounding normal bone tissues (“Normal”) were tested. Ppm1e protein expression (*vs*. Erk1/2) and AMPKα1 phosphorylation (*vs*. total AMPKα1) were quantified (**C**–**D**). *p < 0.05 *vs*. “Normal” group.

First, we tested expression of miR-135b-5p, the Ppm1e-targeting miRNA [19, 20], in human osteoblastoma tissues. As demonstrated, miR-135b-5p expression level was significantly downregulated in osteoblastoma tissues (“Tumor”, *n* = 10), as compared to that in the surrounding normal bone tissues (“Normal”, *n* = 10) (Figure 1A). On the other hand, miR-135b-5p's target, Ppm1e mRNA, was upregulated in osteoblastoma tissues (Figure 1B). Ppm1e protein expression was also higher in osteoblastoma tissues than that in normal bone tissues (blot data were summarized in Figure 1C). As discussed above, Ppm1e is an established AMPKα1 phosphatase [18–20]. Thus, Ppm1e upregulation shall cause AMPKα1 de-phosphorylation. Indeed, the level of p-AMPKα1 (Thr-172) in the osteoblastoma tissues was much lower than that in the normal bone tissues (summarized in Figure 1D).

### Forced-expression of miR-135b-5p silences Ppm1e, causing AMPK activation in human osteoblastoma cells

**Figure 2:**
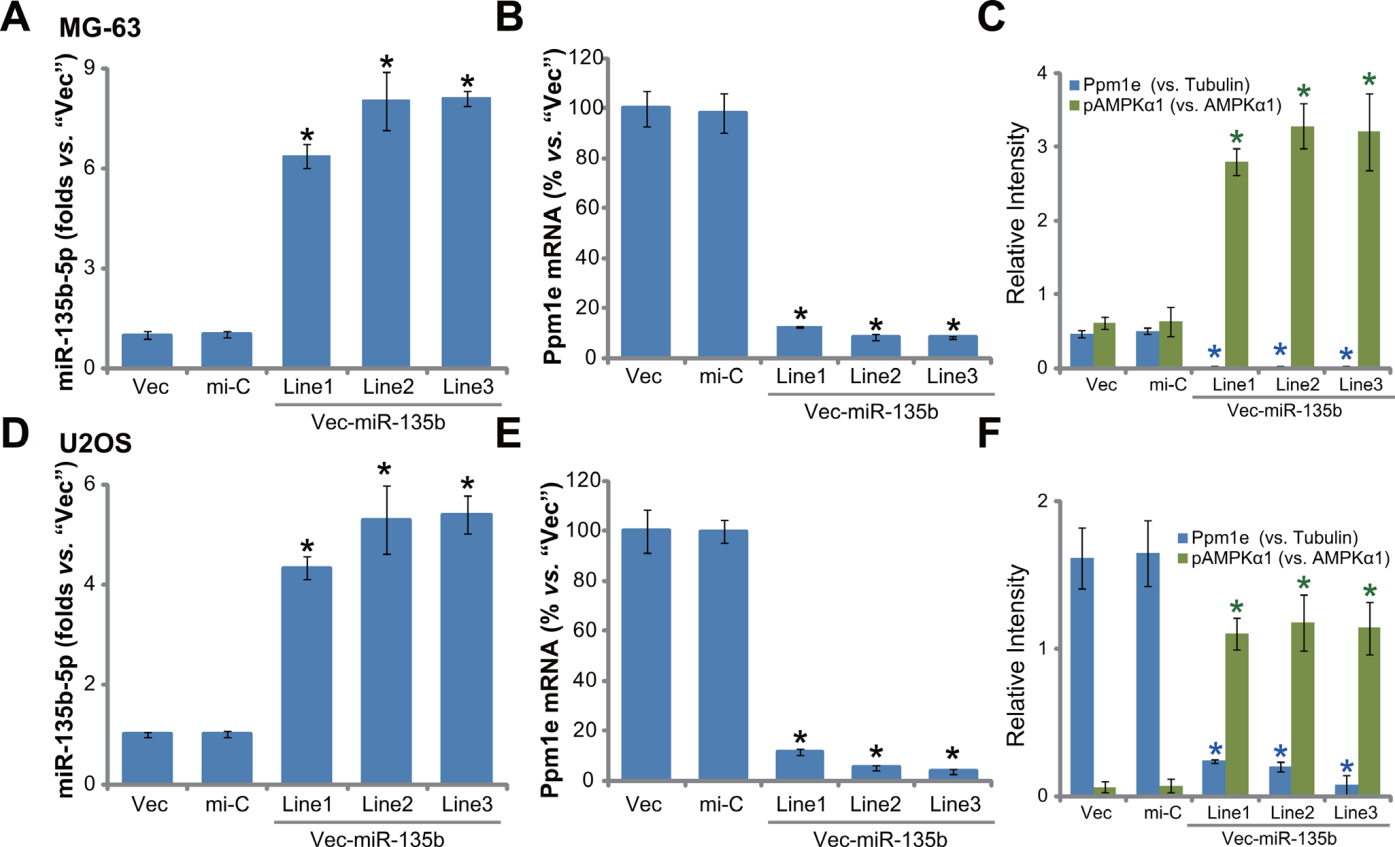
Forced-expression of miR-135b-5p silences Ppm1e, causing AMPK activation in human osteoblastoma cells Stable MG-63 cells (**A**–**C**) or U2OS cells (**D**–**F**), expressing miR-135b vector (“Vec-miR-135b”, three lines each, “L1/L2/L3”), microRNA control (“mi-C”) vector or the empty vector (“Vec”, pSuper-puro), were subjected to qRT-PCR assay to test expression of miR-135b-5p (A and D) and Ppm1e mRNA (B and E); Listed proteins in above cells were tested by Western blot assay, Ppm1e protein expression (*vs*. Tubulin) and AMPKα1 phosphorylation (*vs*. total AMPKα1) were quantified (C and F). For each assay, *n* = 5. *p < 0.05 *vs*. group “mi-C”. Experiments in this figure were repeated three times, and similar results were obtained.

Next, a miR-135b expression vector (“Vec-miR-135b”, a gift from Dr. Cui [19, 20]) was introduced to MG-63 osteoblastoma cells. Via selection by puromycin, a total of three stable MG-63 cell lines (“L1/L2/L3”) expressing Vec-miR-135b were established. qRT-PCR results in Figure 2A demonstrated that expression level of miR-135b-5p was indeed significantly upregulated in the stable cells (“L1/L2/L3”). On the other hand, miR-135b-3p level in above cells was quite low (Data not shown). Consequently, the Ppm1e mRNA was depleted in the three lines (Figure 2B). Protein expression of Ppm1e in these cells was similarly downregulated (Figure 2C), causing AMPK activation or p-AMPKα1 increase (Figure 2C). We also repeated the above experiments in another osteoblastoma cell line, U2OS cells. Once again, forced-expression of miR-135b-5p in three U2OS cell lines (“L1/L2/L3”) (Figure 2D) led to Ppm1e depletion (Figure 2E and 2F) and significant AMPK activation (Figure 2F). Introduction of the non-sense microRNA control (“mi-C”) showed no effect on expressions of miR-135b, Ppm1e and p-AMPKα1 (Figure 2A–2F). Collectively, miR-135b-5p expression causes Ppm1e silence and AMPK activation in human osteoblastoma cells.

### Forced-expression of miR-135b-5p inhibits osteoblastoma cell proliferation

**Figure 3:**
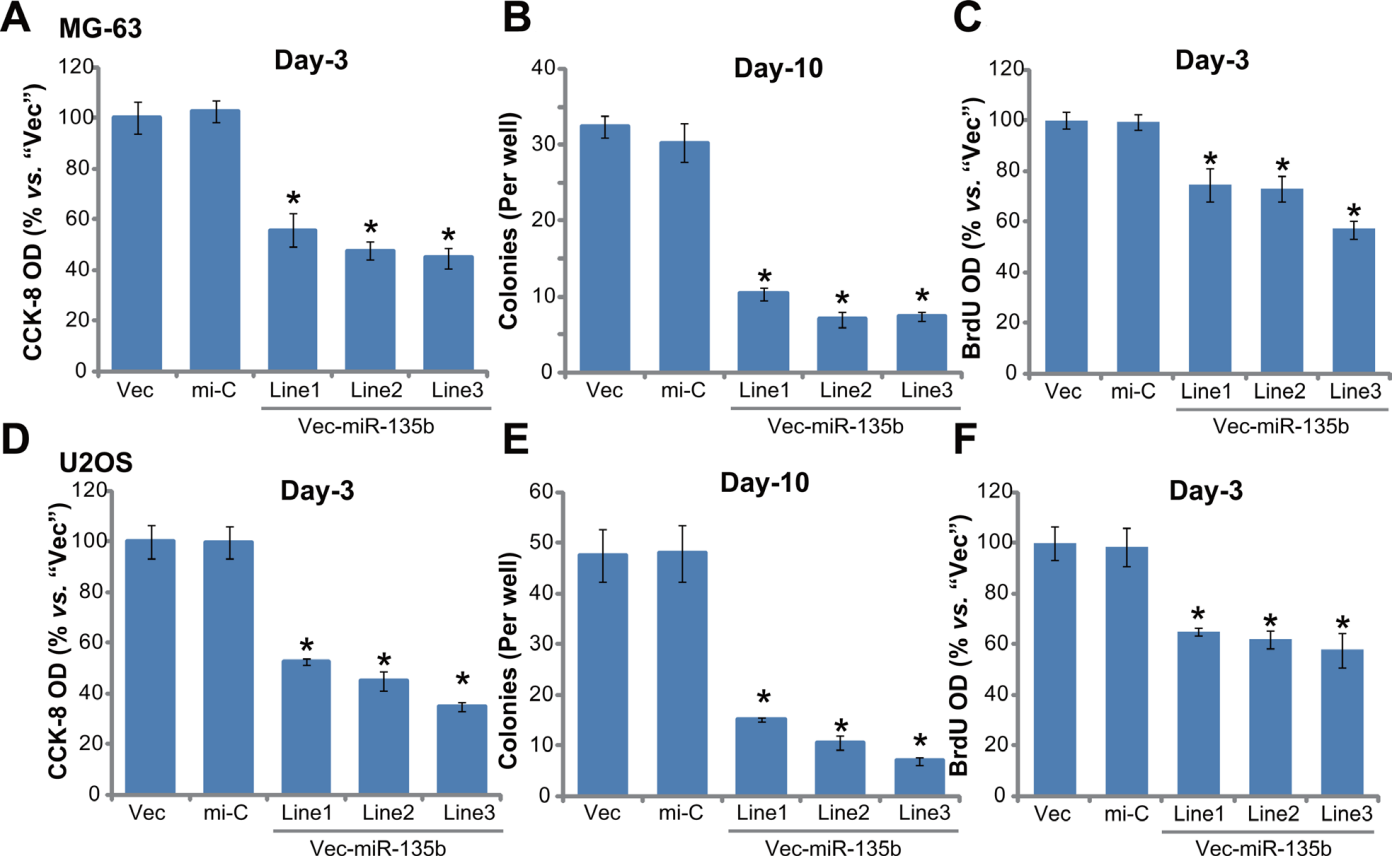
Forced-expression of miR-135b-5p inhibits osteoblastoma cell proliferation Stable MG-63 cells (**A**–**C**) or U2OS cells (**D**–**F**), expressing miR-135b vector (“Vec-miR-135b”, three lines each, “L1/L2/L3”), microRNA control (“mi-C”) vector or the empty vector (“Vec”, pSuper-puro), were subjected to following cell proliferation assays: CCK-8 assay (A and D), colony formation assay (B and E) and BrdU incorporation assay (C and F). Notably, exact same number of viable cells of different background were plated initially for these proliferation assays (Same for all Figures). For each assay, *n* = 5. *p < 0.05 *vs*. group “mi-C”. Experiments in this figure were repeated three times, and similar results were obtained.

Above results confirmed significant AMPK activation in miR-135b-expressed cells. As discussed above, activation of AMPK could possibly inhibit proliferation of many human cancer cells [23–26]. We thus tested the proliferation of the above osteoblastoma cells. Cell Counting Kit-8 (CCK-8) cell proliferation assay results in Figure 3A clearly showed that proliferation of MG-63 cells with miR-135b vector (three lines, “L1/L2/L3”, see Figure 2) was significantly inhibited, as compared to cells with non-sense microRNA control (“mi-C”) or empty vector (Figure 3A). Further, forced-expression of miR-135b-5p in MG-63 cells also dramatically decreased the number of colonies (Figure 3B). BrdU ELISA OD in these miR-135b-5p-expressing cells was also decreased (Figure 3C). Very similar results were also obtained in U2OS cells. In the three lines (“L1/L2/L3”) of miR-135b-5p-expressing U2OS cells, CCK-8 OD (Figure 3D), colony formation (Figure 3E) and BrdU incorporation ELISA OD (Figure 3F) were all decreased. Introduction of the non-sense microRNA control (“mi-C”) had no such effect on the osteoblastoma cells (Figure 3A–3F). These results demonstrate that forced miR-135b-5p expression inhibits osteoblastoma cell proliferation.

### Ppm1e shRNA knockdown activates AMPK and inhibits osteoblastoma cell proliferation

**Figure 4:**
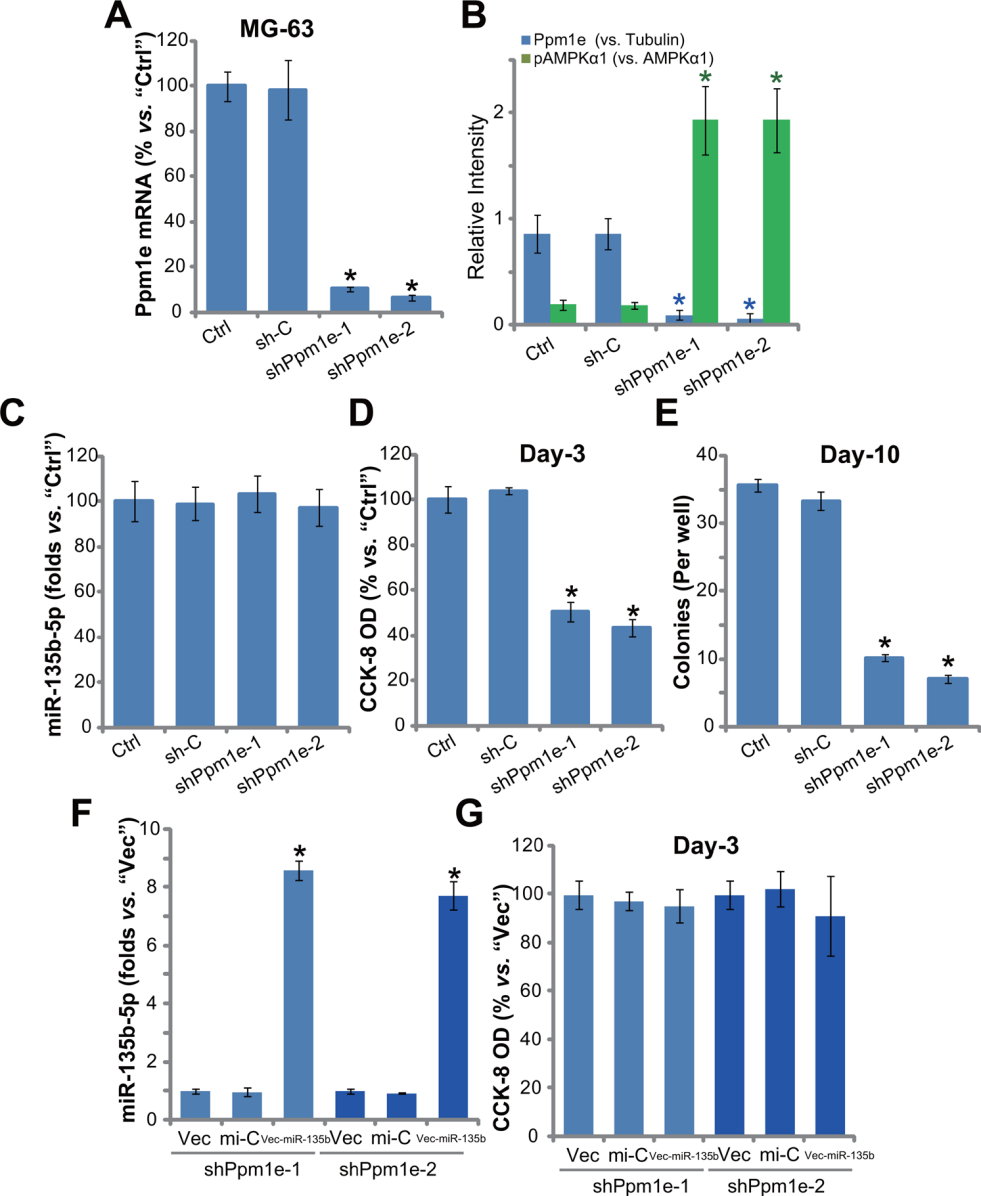
Ppm1e shRNA knockdown activates AMPK and inhibits osteoblastoma cell proliferation Stable MG-63 cells with listed Ppm1e shRNA (“shPpm1e-1” or “shPpm1e-1”) or scramble shRNA control (“sh-C”), as well as the parental control MG-63 cells (“Ctrl”) were subjected to qRT-PCR assay to test expression of Ppm1e mRNA (**A**) and miR-135b-5p (**C**); Listed proteins in above cells were also tested, and blot data were quantified (**B**). Proliferation of above cells was tested by the CCK-8 assay (**D**) and colony formation assay (**E**). MG-63 cells with listed Ppm1e shRNA (“shPpm1e-1” or “shPpm1e-1”) were also transfected with miR-135b vector (“Vec-miR-135b”), expression of miR-135b-5p was tested afterwards (**F**); Cell proliferation was examined by CCK-8 assay (**G**). Ppm1e protein expression (*vs*. Tubulin) and AMPKα1 phosphorylation (*vs*. total AMPKα1) were quantified (B). Notably, exact same number of viable cells of different background were plated initially for these proliferation assays. For each assay, *n* = 5. *p < 0.05 *vs*. group “sh-C”/“mi-C”. Experiments in this figure were repeated three times, and similar results were obtained.

Above results have shown that forced-expression of miR-135b-5p silenced Ppm1e and inhibited osteoblastoma cell proliferation. If Ppm1e is the direct target of miR-135b-5p, knockdown of Ppm1e shall also inhibit osteoblastoma cell proliferation. To test this hypothesis, a panel of two distinct lentiviral Ppm1e shRNAs (“shPpm1e-1” and “shPpm1e-1”, gifts from Dr. Cui's group [20], were employed. Results showed that the applied Ppm1e shRNAs efficiently silenced Ppm1e in MG-63 cells (Figure 4A and 4B). As a result, AMPK activation, or p-AMPKα1, was increased (See quantified results in Figure 4B). Expression of miR-135b-5p, as expected, was unchanged in Ppm1e-silenced cells (Figure 4C). Remarkably, proliferation of MG-63 cells, tested again by the CCK-8 assay (Figure 4D) and colony formation assay (Figure 4E), was inhibited with Ppm1e shRNA knockdown. The scramble shRNA control (“sh-C”) didn't change Ppm1e expression, miR-135b-5p level, AMPK activation and MG-63 cell proliferation (Figure 4A–4E).

Based on the results above, we speculated that miR-135b-5p could possibly be invalid in Ppm1e-depleted cells. Thus, we therefore exogenously expressed miR-135b vector in the two MG-63 lines with Ppm1e shRNA (“shPpm1e-1” and “shPpm1e-1”). As shown in Figure 4F, the level of miR-135b-5p was again elevated after expression of vector. Yet, unlike control cells (See Figure 3), forced-expression of miR-135b-5p failed to further inhibit the proliferation of Ppm1e-silneced cells (Figure 4G). These results together imply that Ppm1e is the direct and primary target of miR-135b-5p in mediating its actions in osteoblastoma cells.

### AMPKα1 shRNA knockdown or mutation abolishes miR-135b-5p's actions against osteoblastoma cells

**Figure 5:**
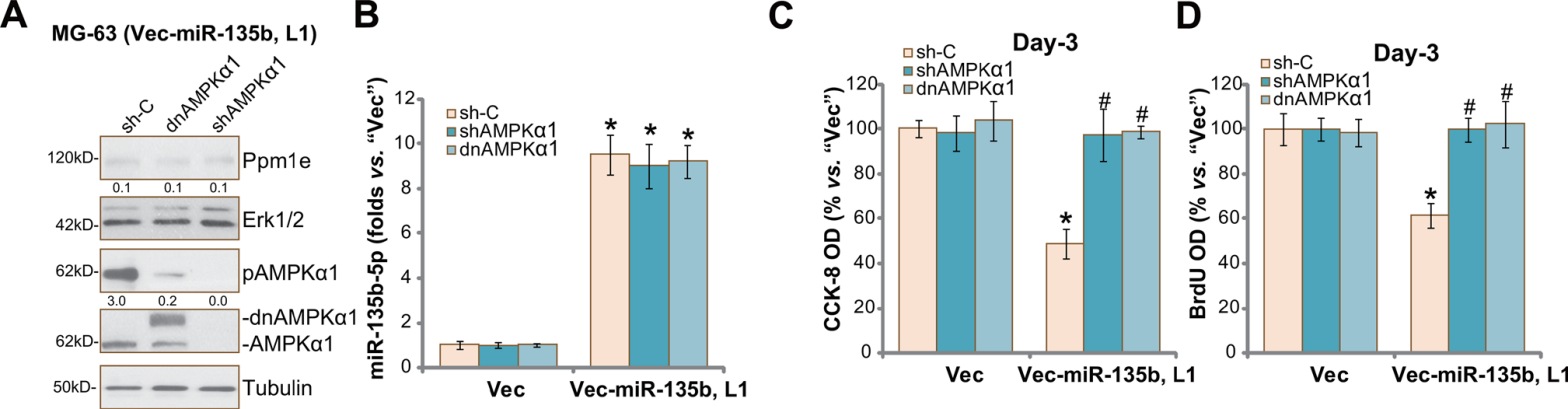
AMPKα1 shRNA knockdown or mutation abolishes miR-135b-5p's actions against osteoblastoma cells Stable MG-63 cells with miR-135b vector (“Vec-miR-135b”, “L1”) or the empty vector (“Vec”, pSuper-puro) were further constructed with AMPKα1 shRNA (“shAMPKα1”), scramble control shRNA (“sh-C”) or a dominant negative mutation of AMPKα1 (T172A, Flag-tagged, ““dnAMPKα1”), expressions of listed proteins were shown (**A**); miR-135b-5p mRNA expression was tested by qRT-PCR assay (**B**); Cell proliferation was tested by the CCK-8 assay (**C**) and BrdU ELISA assay (**D**). Ppm1e protein expression (*vs*. Erk1/2) and AMPKα1 phosphorylation (*vs*. Tubulin) were quantified (A). For each assay, *n* = 5. *p < 0.05 *vs*. group “Vec” only. ^#^*p* < 0.05 *vs*. group “sh-C”. Experiments in this figure were repeated three times, and similar results were obtained.

Above results showed that forced-expression of miR-135b-5p activated AMPK signaling and inhibited osteoblastoma cell proliferation. We thus wanted to know the association between the two. In order to block AMPK activation, AMPKα1 shRNA [20, 27] or a dominant negative mutation of AMPKα1 (T172A, Flag-tagged) [20, 27] was introduced to vec-miR-135b-expressing MG-63 cells (“L1”, see Figure 2). As shown in Figure 5A, AMPKα1 shRNA knockdown or T172A mutation almost completely blocked AMPK activation (p-AMPKα1 at Thr-172) in vec-miR-135b-expressing cells. Ppm1e expression (Figure 5A) and miR-135b-5p level (Figure 5B) were unchanged in AMPKα1-silenced or -mutant cells. Remarkably, miR-135b-5p-induced MG-63 cell proliferation inhibition was almost reversed by AMPKα1 knockdown or mutation (Figure 5C and 5D). These results suggest that activation of AMPK is required for miR-135b-5p-induced MG-63 cell proliferation inhibition. We repeated the experiments in U2OS cells, and similar results were obtained (Data not shown).

### U2OS tumor growth in SCID mice is inhibited after expressing Ppm1e shRNA or miR-135b-5p

**Figure 6:**
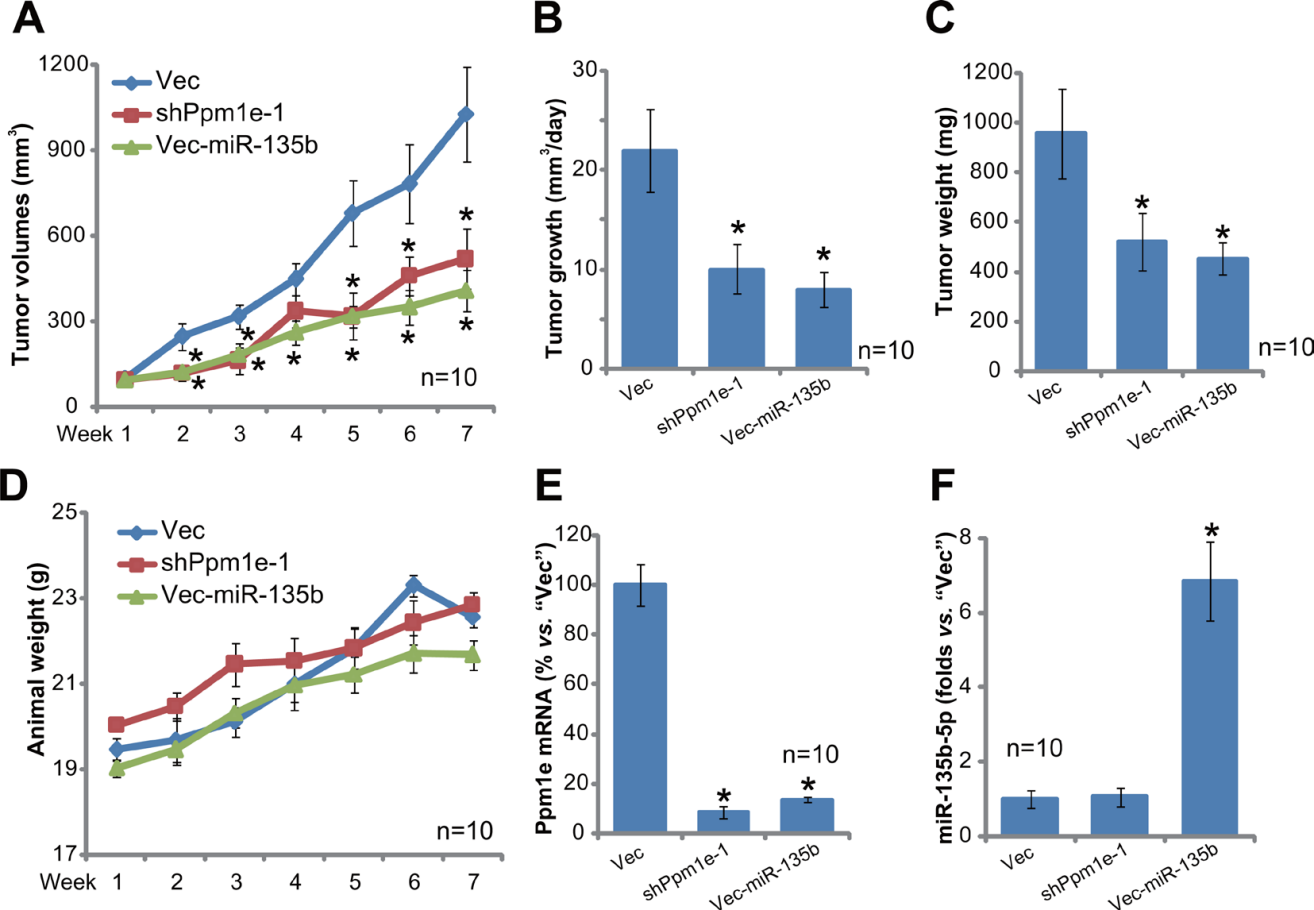
U2OS tumor growth in SCID mice is inhibited after expressing Ppm1e shRNA or miR-135b-5p Stable U2OS cells, with Ppm1e shRNA (“-1”), Vec-miR-135b (“L1”) or empty vector (“Vec”), were inoculated into the SCID mice via *s.c*. injection. When the tumors were around 100 mm^3^ in volume, the recording were started. Tumor volume (**A**) and mice body weight (**D)**, which subtracting estimated tumor weight) were recorded every week for a total of 6 weeks (Week1 to Week7); Estimated daily tumor growth, in mm3 per day, was also calculated (**B**); At week-7, tumors of each group were isolated and weighted (**C**); Expressions of Ppm1e mRNA (**E**), miR-135b-5 (**F**) in tumor tissue lysates were also tested. For each group, *n* = 10. *p < 0.05 *vs*. “Vec” control tumors.

The potential effect of miR-135b-5p on osteoblastoma cell growth *in vivo* was tested. U2OS cells, stably expressing Ppm1e shRNA (“-1”, see Figure 4), Vec-miR-135b (“L1”, see Figure 2) or empty vector (“Vec”, see Figure 2), were inoculated into the severe combined immunodeficient (SCID) mice via *s.c*. injection. Tumor recordings were started when the tumors were around 100 mm^3^ in volume for each group. Tumor growth curve results in Figure 6A demonstrated that growth of U2OS tumors was largely inhibited after expressing Ppm1e shRNA or Vec-miR-135b. Estimated daily tumor growth (in mm^3^ per day) was also significantly lower in Ppm1e shRNA- or Vec-miR-135b-expressing tumors (Figure 6B). At the end of the experiments (Week-7), the weight of tumors with Ppm1e shRNA or Vec-miR-135b was also much lower than “Vec” control tumors (Figure 6C). The mice body weight was indifferent between the three groups (Figure 6D). At Week-7, all xenografted tumors were isolated and lysed. Expressions of miR-135b-5p and Ppm1e were examined. As demonstrated, Ppm1e mRNA was indeed depleted in Ppm1e shRNA- or Vec-miR-135b-expressing tumors (Figure 6E). miR-135b-5p was upregulated in Vec-miR-135b-expressing tumors (Figure 6F). Together, these results indicate that U2OS tumor growth in SCID mice is inhibited after expressing Ppm1e shRNA or miR-135b-5p.

## DISCUSSION

Here, we demonstrated that miR-135b-5p expression was downregulated in human osteoblastoma tissues, which was associated with Ppm1e upregulation. Forced-expression of miR-135b-5p silenced Ppm1e and potently inhibited osteoblastoma cell proliferation *in vitro* and *in vivo*. These results imply that miR-135b-5p could possibly be a novel anti-cancer microRNA (“anti-oncomir”) in osteoblastoma. Remarkably, we imply that AMPK activation could be responsible for miR-135b-5p-mediated anti-osteoblastoma cell activity. In cultured human osteoblastoma cells (MG-63 and U2OS lines), forced-expression of miR-135b depleted Ppm1e, leading to profound AMPK activation (AMPKα1 phosphorylation at Thr-172). Importantly, AMPKα1 shRNA knockdown or dominant negative mutation almost abolished miR-135b-5p-induced actions in osteoblastoma cells.

Intriguingly, we here proposed that Ppm1e is the primary target of miR-135b in mediating its inhibition against osteoblastoma cells. shRNA-mediated knockdown of Ppm1e mimicked miR-135b-5p's actions, and similarly induced AMPK activation and inhibited osteoblastoma cell proliferation. Remarkably, forced-expression of miR-135b-5p failed to further inhibit the proliferation of Ppm1e-silneced cells. Therefore, miR-135b-5p expression was in-effective when Ppm1e was depleted, suggesting that Ppm1e is the direct target of this Anti-oncomir. Notably, we showed that Ppm1e expression was significantly elevated in osteoblastoma tissues. On the other hand, shRNA-mediated Ppm1e knockdown potently inhibited osteoblastoma cell growth *in vivo* and *in vivo*. These results indicate that Ppm1e could be a novel oncotarget protein of osteoblastoma.

It is well-known that AMPK participates in gene transcription, cell mitosis, apoptosis and autophagy [13, 28]. Sustained and profound AMPK activation shall inhibit cell growth, and promote cell death and apoptosis [29]. The cancer-inhibitory activity by AMPK is achieved through regulating one or multiple AMPK downstream signal target proteins, including mTOR inhibition [30–33], p53/p27 activation [34–37], JNK activation [35, 38–40] and Ulk1-autophagy pathway [41]. Indeed, many cytotoxic chemo-agents, including vincristine [36, 42], taxol [39, 43], temozolomide [34], and doxorubicin [30, 35], all activate AMPK signaling to kill cancer cells. Therefore, further studies will be needed to explore the downstream signalings of AMPK that mediate miR-135b's actions in osteoblastoma cells.

## MATERIALS AND METHODS

### Chemicals and reagents

The Ppm1e antibody was provided by Dr. Cui's group at Nantong University [20]. All other antibodies were obtained from Cell Signaling Tech (Denver MA). The enhanced chemiluminescence (ECL) reagents were obtained from Pierce (Rockford, IL). The cell culture reagents were provided by Gibco Co. (Shanghai, China).

### Cell culture

As described [44], osteoblastoma U2OS and MG-63 cells were cultured in DMEM plus FBS medium [6].

### Human tissue specimens

As described previously [5, 6], the surgery-isolated osteoblastoma tissues and surrounding normal bone tissues were separated carefully. Tissues were washed thoroughly and minced into small pieces. Tissues were then mechanically dissociated and lysed by the descried tissue lysis buffer [5, 6]. Tissue lysates were stored in liquid nitrogen. This study was conducted according to the principles expressed in the Declaration of Helsinki. The protocol was approved by Internal Review Board and Ethic Review Board of Soochow University. Written-informed consent was obtained from each participant.

### CCK-8 cell proliferation assay

Cell Counting Kit-8 (CCK-8) (Dojindo, Japan) assay was employed to test cell proliferation according to the attached instructions. The detailed protocol was described in our previous study [44].

### Other cell proliferation assays

Clonogenicity assay of cell growth and BrdU ELISA assay of cell proliferation were described in detail in our previous studies [44, 45].

### Western blotting assay

Cell and tissue lysates (40 μg/sample) were separated by 10–12% SDS-PAGE gel, and were transferred onto PVDF membrane (Millipore, USA). Afterwards, the membrane was blocked, followed by incubation with specific primary and corresponding secondary antibodies. The detection was performed via ECL Supersingnal West Pico Chemiluminescent. Indicated band was quantified via ImageJ software.

### Quantitative real-time PCR assay

The protocol of quantitative real-time reverse transcriptase polymerase chain reaction (“qRT-PCR”) assay was described in detail in our previous studies [6, 44, 45]. We utilized the comparative Ct (2^−ΔΔCt^) method to quantify target mRNA expression [46]. GAPDH was tested as the reference gene [6]. mRNA primers for GAPDH and Ppm1e were provided by Dr. Cui [20]. The expression of mature miR-135b was tested by the TaqMan microRNA assay as described [47]. Ten ng of total RNA per sample was reverse-transcribed via the TaqMan MicroRNA Reverse Transcription Kit (Applied Biosystem, Shanghai, China) [47]. The miR-135b-5p primers were described early [48, 49].

### Forced-expression of miR-135b

The miR-135b pSuper-puro-GFP vector (“Vec-miR-135b”) and miR-control (“miR-C”) vector were provided by Dr. Cui [20]. Cells were transfected with the vector by Lipofectamine 2000 reagent (Invitrogen). After 24 hours, cells were subjected to puromycin (1 μg/mL) selection for another 72 hours. miR-135b-5p expression in the resulting stable cells was tested by qRT-PCR assay.

### shRNA knock and stable cell selection

The two distinct lentiviral Ppm1e shRNAs were provided again by Dr. Cui [20]. The scramble control shRNA (sc-108065) and AMPKα1 shRNA (sc-45312-V) were purchased from Santa Cruz Biotech (Shanghai, China). Osteoblastoma cells were seeded onto six-well plates with 50–60% confluence. Ten μL/mL of lentiviral shRNA was added directly to cultured cells for 24 hours. Afterwards, puromycin (1 μg/mL, Sigma) was added to the culture medium for another 48 hours. The knockdown of targeted protein (Ppm1e or AMPKα1 ) was verified by Western blotting assay and/or qRT-PCR assay.

### AMPK dominant negative mutation

The dominant negative mutant of AMPKα1 (dn-AMPKα1, T172A, Flag-tagged) construct was provided by Dr. Lu [8]. Cells were seeded onto six-well plate with 50–60% confluence. The dn-AMPKα1 vector was transfected to cells using Lipofectamine 2000 protocol [8], and stable cells were selected via puromycin (1 μg/mL, Sigma).

### Mice U2OS xenograft assay

As described [5, 50, 51], CB.17 severe combined immuno-deficient (SCID) male mice (18–20 g) were purchased from the Animal Facility of Soochow University (Suzhou, China). Three million of U2OS cells (per mouse), with Ppm1e shRNA or “Vec-miR-135b”, were inoculated subcutaneously (*s.c*.) into the flanks of the mice. When the xenografts were about 100 mm^3^ in volume, recordings were starte as described [50–52]. The protocols were in accordance with the Institutional Animal Care and Use Committee (IACUC), and were approved by the Ethics Committee and Internal Review Board (IRB) of Soochow University.

### Statistical analysis

The quantitative data presented in this study was mean ± standard deviation (SD). Statistical differences were analyzed by one-way ANOVA with post hoc Bonferroni test.

## CONCLUSIONS

We conclude that miR-135b silences Ppm1e to provoke AMPK activation and inhibit osteoblastoma cell proliferation.
